# Acute Hyperglycemia and Its Impact on Mortality of Acute Coronary Syndrome Patients: A Systematic Review

**DOI:** 10.7759/cureus.66365

**Published:** 2024-08-07

**Authors:** Ravi K Pandey, Bo B Lwin, Apoorva Vashishta, Samreen Nishat, Isaac N Mueka, Maria U Hassan, Marcellina Nwosu

**Affiliations:** 1 Clinical Research, California Institute of Behavioral Neurosciences & Psychology, Fairfield, USA

**Keywords:** stemi, unstable angina, nstemi, heart attack, adverse outcome, prognosis, mortality, admission hyperglycemia, stress hyperglycemia, acute hyperglycemia

## Abstract

Acute hyperglycemia or stress hyperglycemia is a frequent finding in patients with acute coronary syndrome (ACS). Several studies have demonstrated the association between acute hyperglycemia with short- and long-term mortality in ACS patients. But the evidence is not concrete. We gathered 1056 articles from three databases, i.e., PubMed, Google Scholar, and Science Direct using different search strategies and filters. We then removed duplicates and 919 articles were screened with title abstract and full text. After a full-text screening of 169 articles, we removed 116 articles. We then applied eligibility criteria and did a quality assessment of articles and finally, we included 21 articles in our study. The 21 articles spanned years 2014 to 2024. Of them, 16 articles were observational studies, two were systematic reviews and meta-analyses, and three were review articles. Six articles used stress hyperglycemia ratio (SHR) alone, seven articles used admission blood glucose (ABG) alone, two used fasting plasma glucose (FPG) alone and one used SHR, ABG, and FPG together as a parameter to measure acute hyperglycemia. Short-term poor outcomes (in-hospital, <30 days) were studied in 12 studies, and long-term poor outcomes (>30 days-1 year, >1 year) were studied in six studies. A positive correlation between acute hyperglycemia and short- and long-term mortality was found in our 21 included studies. The three parameters which are used to quantify acute or stress hyperglycemia in our study, i.e., SHR, ABG, and FPG predict both short- and long-term mortality in ACS patients. Further study is needed to determine the accurate cutoff level of hyperglycemia to be called acute hyperglycemia in diabetics. We tried to review the recent literature on this topic to deepen our understanding of this topic and to provide a base for future research.

## Introduction and background

Acute coronary syndrome (ACS) is a common cause of morbidity and mortality from cardiovascular causes worldwide in the present era [1-3]. ACS includes two pathologies, i.e., ST elevation-ACS and non-ST elevation-ACS. Non-ST elevation-ACS includes two entities, i.e., unstable angina and NSTEMI (non-ST elevation myocardial infarction). Acute hyperglycemia is a commonly encountered condition in ACS due to neuro-hormonal factors and other factors like beta cell dysfunction, cytokines, and insulin resistance. This acute hyperglycemia or stress hyperglycemia is a strong indicator of poor prognosis in ACS patients by mechanisms not properly elucidated [1-5].

To date, acute hyperglycemia has been quantified using several parameters like stress hyperglycemia ratio (SHR), admission blood glucose (ABG), fasting plasma glucose (FPG), ABG to glycated albumin index and triglyceride glucose index. Studies have suggested that acute hyperglycemia quantified by SHR, ABG, or FPG is associated with increased in-hospital and after-discharge mortality irrespective of diabetic status [3,6-12]. A complicated interplay of neurohormonal factors and cytokines plays a role in the development of acute hyperglycemia in ACS and then the hyperglycemia through various pathologic mechanisms leads to increased complications in ACS patients [13-21].

The accurate pathophysiology by which acute hyperglycemia leads to mortality in ACS patients is not elucidated [3-10]. Also, the precise cutoff to call acute hyperglycemia in diabetic and nondiabetic is not specific and varies from study to study. Gaining glucose levels in control through appropriate management strategies can improve outcomes by improving endothelial function, reducing inflammatory and clotting mediators, and reducing infarct size [20]. The multifactorial pathogenesis of acute hyperglycemia has brought the need for multiple therapeutic strategies that counteract the factors involved in the pathogenesis of hyperglycemia to improve overall outcomes in ACS patients [20]. In this systematic review, we tried to review the studies that have been done in recent years showing the association between acute hyperglycemia and mortality in ACS patients to provide up-to-date evidence regarding this association so that it could broaden our current knowledge on this topic and provide a base for future research.

## Review

Method

This review has been written following Preferred Reporting Items for Systematic Review and Meta-Analyses (PRISMA) [22].

Eligibility Criteria

We included observational studies, systematic reviews, meta-analyses, and review articles. Articles that were in English and studied all-cause and cardiovascular mortality were included. Studies conducted in humans with age of patients above 18 years were included. Book chapters, letters to the editor, case reports, and case series were excluded. Similarly, articles written in a language other than English and articles measuring effects other than mortality were excluded. Parameters like SHR, ABG, and FPG used to quantify acute or stress hyperglycemia were included. Articles using triglyceride glucose index at admission and glucose to glycated albumin ratio to measure acute hyperglycemia were excluded. 

Databases and Search Strategy

The literature search was performed in April 2024. We searched three databases; PubMed, Google Scholar, and Science Direct. For PubMed, we used regular search, MeSH (Medical Subject Headings) search, and used filters. For Google Scholar and Science Direct, we used the regular keyword search using Boolean and filters. We selected only the articles relevant to our topic from Google Scholar and Science Direct. We transferred the gathered articles from the three databases to Endnote (Clarivate, London, UK) and from Endnote to Microsoft Excel (Microsoft Corporation, Redmond, WA, USA). Then we removed 137 duplicates. Then we went for screening and quality check. A detailed MeSH search strategy has been presented in Table 1 below.

**Table 1:** Databases and search strategy. ACS: acute coronary syndrome, STEMI: ST elevation myocardial infarction, NSTEMI: non-ST elevation myocardial infarction, CAD: coronary artery disease, MeSH: medical subject headings. Boolean includes AND, OR, and NOT.

Database	Date of search	Keyword used	Search strategy	Filters	Articles retrieved
PubMed	19/04/2024	Acute hyperglycemia AND mortality AND ACS patients	Regular search with keywords using Boolean	No	174
PubMed	19/04/2024	Keywords-; Acute hyperglycemia, admission hyperglycemia, new onset hyperglycemia, stress hyperglycemia mortality, adverse event, outcome, poor prognosis, ACS, STEMI, NSTEMI, heart attack, CAD. Concept 1-: Hyperglycemia OR raised blood glucose OR stress hyperglycemia OR admission hyperglycemia OR new onset hyperglycemia OR ("Hyperglycemia/complications"[Majr] Or "Hyperglycemia/etiology"[Majr] OR "Hyperglycemia/mortality"[Majr] OR "Hyperglycemia/physiopathology"[Majr]). Concept 2-: Mortality or poor prognosis OR adverse outcome OR adverse cardio vascular effect OR "Mortality"[Majr]. Concept 3-: ACS OR CAD OR STEMI OR NSTEMI OR unstable angina OR heart attack OR ("Acute Coronary Syndrome/mortality"[Majr] OR "Acute Coronary Syndrome/physiopathology"[Majr])	Building block technique using Boolean search and MeSH #1 AND #2 AND #3-: Hyperglycemia OR raised blood glucose OR stress hyperglycemia OR admission hyperglycemia OR new onset hyperglycemia OR ("Hyperglycemia/complications"[Majr] Or "Hyperglycemia/etiology"[Majr] OR "Hyperglycemia/mortality"[Majr] OR "Hyperglycemia/physiopathology"[Majr]) AND Mortality or poor prognosis OR adverse outcome OR adverse cardio vascular effect OR "Mortality"[Majr] AND ACS OR CAD OR STEMI OR NSTEMI OR unstable angina OR heart attack OR ("Acute Coronary Syndrome/mortality"[Majr] OR "Acute Coronary Syndrome/physiopathology"[Majr])	No	71602
PubMed	19/04/2024	Concept 1-: Hyperglycemia OR raised blood glucose OR stress hyperglycemia OR admission hyperglycemia OR new onset hyperglycemia OR ("Hyperglycemia/complications"[Majr] Or "Hyperglycemia/etiology"[Majr] OR "Hyperglycemia/mortality"[Majr] OR "Hyperglycemia/physiopathology"[Majr]). Concept 2-: Mortality or poor prognosis OR adverse outcome OR adverse cardio vascular effect OR "Mortality"[Majr]. Concept 3-: ACS OR CAD OR STEMI OR NSTEMI OR unstable angina OR heart attack OR ("Acute Coronary Syndrome/mortality"[Majr] OR "Acute Coronary Syndrome/physiopathology"[Majr])	Building block technique using Boolean search and MeSH #1 AND #2 AND #3-: Hyperglycemia OR raised blood glucose OR stress hyperglycemia OR admission hyperglycemia OR new onset hyperglycemia OR ("Hyperglycemia/complications"[Majr] Or "Hyperglycemia/etiology"[Majr] OR "Hyperglycemia/mortality"[Majr] OR "Hyperglycemia/physiopathology"[Majr]) AND Mortality or poor prognosis OR adverse outcome OR adverse cardio vascular effect OR "Mortality"[Majr] AND ACS OR CAD OR STEMI OR NSTEMI OR unstable angina OR heart attack OR ("Acute Coronary Syndrome/mortality"[Majr] OR "Acute Coronary Syndrome/physiopathology"[Majr])	< 10 year, Observational studies, review articles, meta-analysis, Free full text, English language, only humans	963
Google Scholar	14/04/2024	Acute hyperglycemia AND mortality AND ACS	Regular search using keyword and Boolean	No	40600
	14/04/2024	Acute hyperglycemia AND mortality AND ACS	Regular search using keyword and Boolean	Year- 2014-2024	1782
	14/04/2024	Acute hyperglycemia AND mortality AND ACS	Regular search using keyword and Boolean	Articles relevant to topic	76
Science Direct	14/04/2024	Acute hyperglycemia AND mortality AND ACS	Regular search using keyword and Boolean	No	6004
	14/04/2024	Acute hyperglycemia AND mortality AND ACS	Regular search using keyword and Boolean	English, Medicine and dentistry, original article and review, articles<10 years	970
	14/04/2024	Acute hyperglycemia AND mortality AND ACS	Regular search using keyword and Boolean	Articles relevant to topic	17

Result

Study Selection

**Figure 1:**
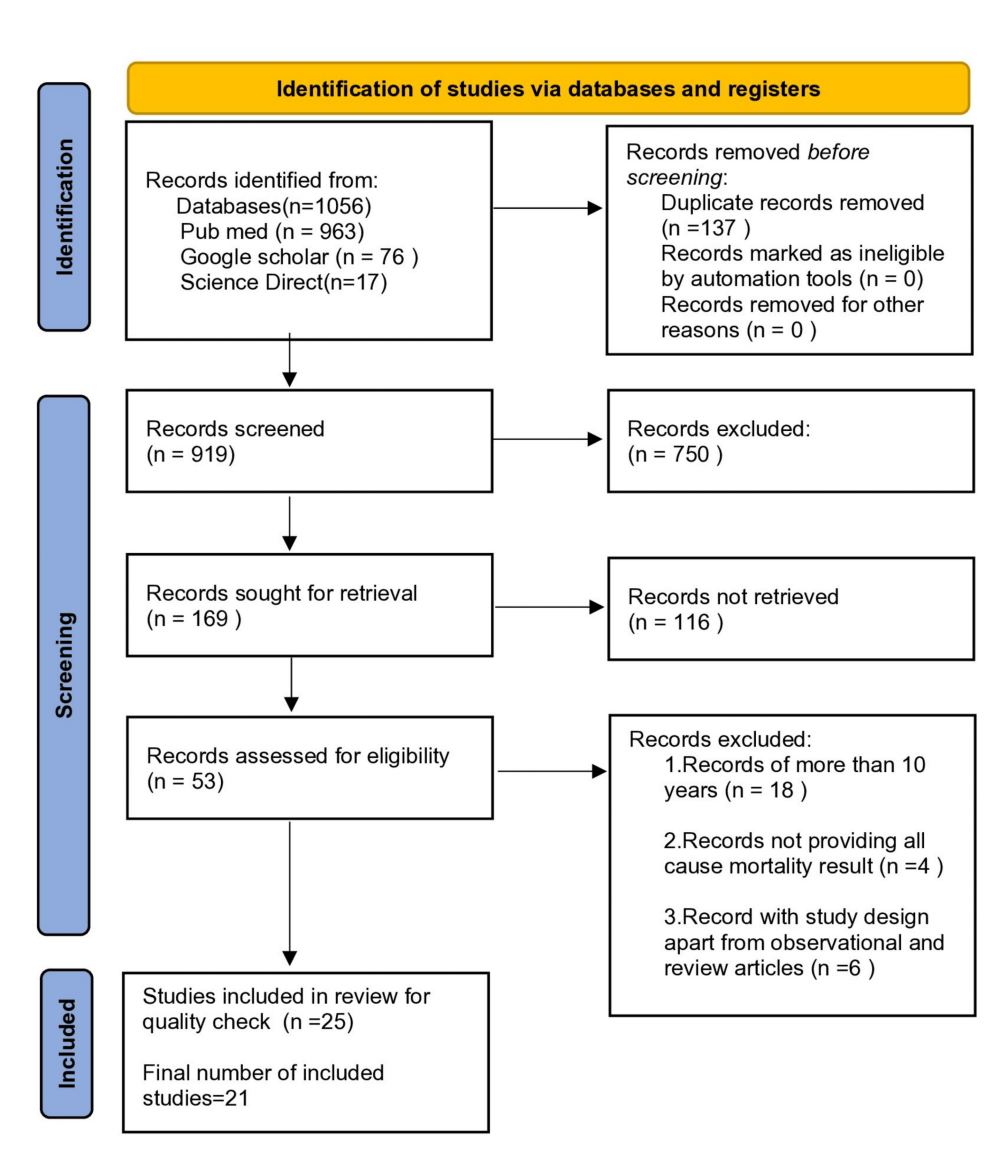
PRISMA flow diagram. PRISMA: Preferred Reporting Items for Systematic Review and Meta-Analyses.

We gathered 1056 articles from the three databases PubMed, Google Scholar, and Science Direct. Among them, 137 duplicates were removed. Then 919 articles were screened with titles and abstracts. In all, 750 articles were excluded and 169 articles were screened with full-text reading. After full-text reading, 116 articles were excluded. Then we applied eligibility criteria to the remaining 53 articles after which 28 articles were removed. Finally, 25 articles were included for quality check. Four articles were removed because of the high risk of bias. A PRISMA flow diagram displaying the number of articles from identification to the number of articles collected for review is presented in Figure 1 below [23].

Study Characteristics

We have included 21 studies in our systematic review after quality check. The publication year of the articles ranges from 2014-2024. The 21 articles included consist of 16 observational studies, three review articles, and two systematic reviews and meta-analyses. Among observational studies, six were retrospective cohort studies, 11 were prospective cohort studies, and one was a cross-sectional study. One study was a systematic review and meta-analysis and one was a dose-response meta-analysis [1,3]. Two observational studies included ACS with comorbidities like triple vessel diseases (TVD) and patients on dialysis [14,17]. Hyperglycemia was quantified using different parameters; seven studies used ABG, six studies used SHR, two studies used FPG, and one study used ABG, SHR, and FPG together. Studies used different follow-up periods. We divided the short-term follow-up into in-hospital and ≤ 30 days follow-up and long-term follow-up into 30 days to ≤ 1 year or > 1-year follow-up. A study characteristics table including author name, date of publication, total participants in each study, follow-up period, mean age of participants, hyperglycemia parameters and cutoffs of each study, glucometabolic status, result, and conclusion of each study is presented below in Tables 2, 3.

**Table 2:** Study characteristics table. ACS: acute coronary syndrome, STEMI: ST-elevation myocardial infarction, NSTEMI: non-ST elevation myocardial infarction, DM: diabetes mellitus, SHR: stress hyperglycemia ratio, PCI: percutaneous coronary intervention, ABG: admission blood glucose, FPG: fasting plasma glucose, OR: odds ratio, RR: relative risk, CI: confidence interval, AMI: acute myocardial infarction, RCS: restricted cubic spline, ROC: receiver operating characteristics curve, NSTE-ACS: non-ST elevation acute coronary syndrome, RPG: random plasma glucose, HR: hazard ratio, HbA1c: glycosylated hemoglobin. Alkatiri et al. [1], Chen et al. [2], Cheng et al. [3], Ding et al. [4], Gencer et al. [5], Kumar et al. [6], and Liu et al. [7]. [11]

First author name	Year of publication	Study design	Total number of patients and population characteristics	Recruitment period	Follow-up duration	Mean age	Glucometabolic status	Hyperglycemia definition and cutoff	Result	Conclusion
Alkatiri et al.	2024	Systematic review and meta-analysis	164937 STEMI Patients	Articles from 2005-2023	In-hospital mortality and 30 days mortality	58.5 years	1/3^rd^ had history of DM	The blood glucose level cutoff for hyperglycemia was 110 mg/dl. Hyperglycemia was defined based on admission blood glucose, HbA1c and fasting plasma glucose [1].	Admission hyperglycemia predicted all-cause mortality at different follow-up length. i.e., in-hospital (RR 3.41, 95% CI: 2.82–4.14), at 30 days (RR 3.71, 95% CI: 2.95–4.66), at 6 months (RR 1.80, 95% CI: 0.96–3.36), and >6 months of follow-up (RR 2.32, 95% CI: 1.90–2.82) [1].	Hyperglycemia predict poor outcome in STEMI patients. ABG and FPG levels predict short-term poor outcome in STEMI patients, whereas HbA1c may be a more suitable predictor of long-term poor outcome [1].
Chen et al.	2021	Retrospective cohort	341 AMI patients aged more than 45	January 1, 2014 to December 31, 2019	In-hospital mortality	80.67 ± 4.10 years	100 had DM	Hyperglycemia was measured using SHR. Patients were divided into two groups, the low SHR group (<1.25 SHR, n = 208) and high SHR group (>1.25 SHR, n = 133) [2].	In-hospital all-cause mortality in low SHR was 17/208 and in high SHR was 27/133. A high SHR was positively corelated with higher risk of in-hospital mortality (OR = 2.862, 95% CI = 1.492–5.491, P = 0.002); and the association remained persistent after adjusting for potential confounding factors also (OR: 2.871, 95% CI: 1.428–5.772, p = 0.003). When used as a continuous variable, SHR remained an independent predictor of in-hospital all-cause death (OR: 2.513, 95% CI: 1.153–5.476, p = 0.020) in multivariate analysis [2].	High SHR is associated with increased mortality in AMI patients especially in non-diabetic population [2].
Cheng et al.	2021	Dose response meta-analysis	47177 AMI in adult aged >18	Inception to first April 2022	In-hospital mortality, less than one year and more than one year mortality	>18 years	24 studies included cases with DM	Admission hyperglycemia was defined as blood glucose levels > 140 mg/dl (7.8 mmol/L) at admission [3].	Admission hyperglycemia was associated with an increased risk of all-cause mortality (relative risk: 3.12, 95% confidence interval 2.42–4.02) in a short follow-up. In long-term follow-up also, admission hyperglycemia was associated with an heightened risk of all-cause mortality (1.97, 1.61–2.41) [3].	High admission blood glucose was associated with higher poor outcome risk in both diabetic and non-diabetic [3].
Ding et al.	2019	Retrospective cohort	1698 AMI patients without DM	January 2013 to March 2018	In-hospital mortality	64.33 years	No DM cases	Hyperglycemia was quantified by ABG. Patients were categorised in to three groups based on ABG as follows: group 1 euglycemic group, admission glucose levels ≤140 mg/dL ( n = 1216); group 2 moderate hyperglycemia group, admission glucose levels > 140 and < 180 mg/dL (n = 370); and group 3 severe hyperglycemia group, admission glucose levels ≥180 mg/dL (n = 112) [4].	The in-hospital all-cause mortality risk comparisons between groups 3 and 1 and groups 3 and 2 were statistically significant (OR = 4.595, P<0.001 and OR=3.873, P = 0.006 respectively. More mortality was seen in group 2 and group 3 [4].	All-cause in-hospital mortality increased in AMI patients on increase in ABG level especially when admission glucose levels ≥180 [4].
Gencer et al.	2020	Prospective cohort	3858 ACS patients aged > 18	December 2009-December 2014	1 year	Non-diabetic-62.4±12.2 years vs diabetic 66.3±11.7 years	709 DM cases	Hyperglycemia was quantified using FPG. Hyperglycemia was defined as fasting plasma glucose ⩾10 mmol/l recorded within the first 24 h of hospital admission [5].	A positive corelation was found between hyperglycemia and all-cause death at one year in non-diabetic patients with an unadjusted HR of 2.53 (95% CI 1.10–5.83, p=0.03). The association remained still pertinent in multivariate analysis (adjusted HR 2.39; 95% CI 1.03–5.56, p=0.04). Univariate analysis of diabetic for all-cause mortality was 1.87 (1.22–2.86) p=0.004 [5].	Fasting hyperglycemia of ⩾10 mmol/l at presentation predicted higher one-year mortality in non-diabetic patients [5].
Kumar et al.	2023	Prospective cohort	4470 STEMI patients undergoing primary PCI	January 2022 to June 2022	In-hospital mortality	55.52 ± 11 years	1037 patients with DM	Acute hyperglycemia was defined as Random Plasma Glucose (RPG) > 200 mg/dl at the time of presentation to the emergency room [6].	Acute hyperglycemia was found to be a significant predictor of mortality with an adjusted OR of 1.81 (95% CI: 1.28–2.55) in multivariable analysis [6].	Higher blood glucose is associated with heightened risk of in-hospital mortality [6].
Liu et al.	2021	Retrospective cohort	498 NSTE-ACS	March 2018 and November 2020	In-hospital mortality	A = 65 ± 8 years B = 68 ±7 years C = 76 ± 6 years	No DM patient	Based on the admission blood glucose level, NSTE-ACS patients were divided into three groups: A (BG < 7.8 mmol/L), B(7.8 mmol/L ≤ BG < 11.1 mmol/L), and C (BG ≥ 11.1 mmol/L) [7].	Higher ABG increase risk of in-hospital mortality with OR =1.81(1.26-2.41) p<0.01 on multivariable analysis [7].	Admission hyperglycemia was associated with adverse outcomes in NSTE-ACS patients [7].

**Table 3:** Study characteristics table. MIOCA: myocardial infarction with obstructive coronary artery, MINOCA: myocardial infarction with non-obstructive coronary artery, aHGL: admission hyperglycemia, ACS: acute coronary syndrome, STEMI: ST elevation myocardial infarction, NSTEMI: non-ST elevation myocardial infarction, DM: diabetes mellitus, SHR: stress hyperglycemia ratio, ABGF: admission blood glucose, FPG: fasting plasma glucose, OR: odds ratio, RR: relative risk, CI: confidence interval, AMI: acute myocardial infarction, RCS: restricted cubic spline, ROC: receiver operating characteristics curve, NSTE-ACS: non-ST elevation acute coronary syndrome, RPG: random plasma glucose, AUC: area under curve, HR: hazard ratio, TVD: triple vessel diseases, HbA1c: glycosylated hemoglobin, CAD: coronary artery disease. Paolisso et al. [8], Schmitz et al. [9], Shahid et al. [10], Thoegersen et al. [11], Upur et al. [12], Wei et al. [13], Xie et al. [14], Xu et al. [15], Zeng et al. [16], Zhang et al. [17], and Zhao et al. [18].

First author name	Year of publication	Type of study	Total participants and its characteristics	Recruitment period	Follow-up duration	Mean age	Glucometabolic status	Hyperglycemia definition and cutoff	Result	Conclusion
Paolisso et al.	2021	Prospective cohort	2431 with AMI MIOCA-; 2198 MINOCA-; 233	January 2016-September 2020	In-hospital death and > 1 year mortality	MIOCA- No aHGL-; median age 69 years, aHGL-; median age 73 years MINOCA-No aHGL-; median age 68 years, aHGL-; median age 74 years	604 patients with DM	Admission blood glucose level more than equal to 140 was used to classify patient in to admission hyperglycemia (aHGL) and nonhyperglycemic (non aHGL) [8].	In-hospital mortality was significantly higher in aHGL patients (4.6% vs 0.8%, p <0.001) in the MIOCA group. 283 deaths were recorded over the period of 26 months. All-cause mortality was higher in hyperglycemic group (17.2% vs 8.9%, p<0.001 and 22.9% vs 7.7%, p=0.006) compared to euglycemic in both MIOCA and MINOCA. Both in MIOCA and MINOCA group patients, cardiovascular deaths were observed more in hyperglycemic patients (10.1% vs %5.1% p<0.001 and 14.3 vs 3.6% p=0.009, respectively) [8].	aHGL was identified as an independent predictor of worse short- and long-term outcomes in both MIOCA and MINOCA patients irrespective of diabetes status [8].
Schimitz et al.	2022	Prospective cohort	2311 AMI patients aged 25-84	2009-2014	28 day and > 1 year mortality	DM- Mean age 68 ±11 years, Non-DM–mean age 63.7±12.7 years	681 patients with DM	Hyperglycemia was quantified by SHR. SHR was calculated using standard formula [9].	Higher ABG and higher SHR predicted higher 28 days mortality in both diabetic and non-diabetics. Admission glucose predicted the short-term mortality significantly better than SHR area under curve (AUC) SHR: 0.6912 {95%CI 0.6317–0.7496}, AUC admission Glucose: 0.716 {95% CI 0.6572–0.7736}, p-value: 0.0351) in non-DM patients. AUC for SHR and ABG were similar in diabetics [9].	Stress hyperglycemia in AMI patients predicts short-term worse outcome better than long-term outcome [9].
Shahid et al.	2020	Prospective cohort	256 STEMI patients older than 18 years	From June 13, 2018 to October 12, 2019	In-hospital mortality	55 ± 11 years	92 patients had DM	Patients were divided into two groups based on glucose levels on admission: a hyperglycemia group (with blood glucose >140 mg/dl) and a euglycemic group (with blood glucose <140 mg/dl) [10].	As a whole in-hospital mortality was higher in the hyperglycemic group (n = 12, 12.5%) than in the euglycemic group (n = 6, 3.7%; p = 0.015) [10].	Admission hyperglycemia is better predictor of death in diabetics than non-diabetics [10].
Thoegersan et al.	2020	Prospective Cohort study	1284 patients with AMI	2010 to 2017	30 day	No DM= 66.9 ±12.2 years, DM1=64.67 ±10.38 years and DM2=69.19±9.95 years	46 DM1, 273 DM2 patients	Hyperglycemia was quantified using ABG. ABG was defined as the first measured glucose level in <2 hours from admission to ICU. DM was defined as admission blood glucose level>9mmol/L [11].	Patients with diabetes had elevated 30-day mortality in comparison with patients without diabetes (62% vs. 50%, *P* =0.001). Increasing admission glucose was associated with increasing 30-day mortality in a dose-dependent manner in diabetes mellitus (4-8 mmol/L, 41%; 8-12 mmol/L, 49%; 12-16 mmol/L, 63%; >16 mmol/L, 67%; *P* = 0.028) and non-diabetes patients (4-8 mmol/L, 32%; 8-12 mmol/L, 43%; 12-16 mmol/L, 57%; >16 mmol/l; 68%; *P* < 0.001) [11].	High admission blood glucose was associated with higher 30 days mortality in both diabetic and non-diabetics [11].
Upur et al.	2022	Retrospective cohort	3330 with AMI	July 2012 and July 2020	>1 year mortality	56.3±12.3 years	1060 patients with DM	Fasting glucose level were measured in all patients. Acute hyperglycemia was defined as FPG levels ≥ 5.6 mmol/L for non-diabetic patients and ≥ 10.6 mmol/L for diabetic patients [12].	Hyperglycemia (non-diabetic patients: HR 2.26, 95% CI 1.35–3.79; diabetic patients: HR 1.70, 95% CI 1.16–2.49) was a significant predictor of short-term mortality (in-hospital death) in univariate cox model. Also, for the long-term outcome, hyperglycemia (non-diabetic patients: HR 1.23; 95% CI 1.01–1.49; diabetic patients: HR 1.23, 95% CI 1.00–1.58) was identified as a predictor of long-term mortality [12].	Admission hyperglycemia was associated with poor short-and long-term outcomes in AMI patients, with or without diabetes [12].
Wei et al.	2023	Retrospective cohort	1099 STEMI patients who underwent PCI	2016 to 2021	>1 year mortality	62.55 ± 13.58 years	297 patients with DM	Hyperglycemia was measured using three parameters, i.e., SHR, ABG and FPG. SHR values were divided in three groups, i.e, (1) SHR G1 was obtained by dividing ABG by the estimated average glucose level. (2) SHR G2, which was obtained by dividing FPG by the HbA1c level. (3) SHR G3, which was obtained by dividing FPG by the estimated average glucose level. The estimated average glucose level was calculated using the following formula: estimated average glucose (mmol/L) = 1.59 × HbA1c (%) − 2.59. The participants were divided into four groups based on admission blood glucose (ABG): group 1 (n = 274), ABG ≤ 6.78mmol/L; group 2 (n = 274), 6.78mmol/L < ABG ≤ 8.02 mmol/L; group 3 (n=276), 8.02 mmol/L < ABG ≤ 10.81 mmol/L; and group 4 (n=275), ABG>10.81 mmol/L [13].	A positive correlation between hyperglycemia status i.e ABG, FPG, and stress hyperglycemia ratios (SHR G1, SHR G2, SHR G3), and heightened risk of in-hospital mortality (ABG OR: 1.27, 95% CI: 1.19–1.36; FBS OR: 1.25, 95% CI: 1.16–1.35; SHR G1 OR: 1.61, 95% CI: 1.21–2.14; SHR G2 OR: 1.57, 95% CI: 1.22–2.01; SHR G3 OR: 1.59, 95% CI: 1.24–2.05) [13].	Results suggested a strong positive association between stress hyperglycemia and heightened risk of in-hospital all-cause mortality, regardless of diabetes status. The relationship between stress hyperglycemia and mortality outcome was nonlinear J- shaped [13].
Xie et al.	2023	Prospective cohort	714 dialysis patients with ACS	January 2015 to June 2021	>1 year mortality	62 years	450 patients with DM	Hyperglycemia was quantified with ABG. ABG was defined as serum glucose measurement taken within 24 h of hospital admission. SHR was determined by standard formula [14].	280 cases of all-cause mortality were observed. When the highest SHR group was compared to the second SHR group, a heightened increased risk of all-cause mortality was observed (adjusted HR, 2.19; 95% CI, 1.64–2.93). Moreover, a non-linear and J-shaped association between the SHR and all-cause mortality (all P values for nonlinearity <0.05) was found on RCS analysis [14].	High SHR is associated with worse long-term outcome in dialysis patients with ACS [14].
Xu et al.	2022	Retrospective cohort	19929 CAD patients including ACS patients also	January 1, 2016 to December 30, 2021	In-hospital mortality	68 ± 11 years	Pre-DM = 2389, DM = 4412	The admission blood glucose (ABG) was measured as the first-measured random serum glucose in <24 h of admission. SHR was calculated using the standard formula. The patients were divided into three groups according to the tertiles of SHR: T1 group (SHR <0.725, n=2732), T2 group (0.725 ≤ SHR<0.832, n=2730), and T3 group (SHR ≥ 0.832, n=2734) [15].	SHR was a strong predictor for in-hospital mortality in ACS group (OR = 19.351; 95% CI = 8.397–44.592; P <0.001) in multivariate analysis. The T3 group in patients with ACS had an heightened risk of in-hospital mortality by 7.9-fold than that of the T1 group (OR = 7.939; 95% CI = 2.723–23.151; P <0.001) [15].	High SHR predicted higher in-hospital mortality in CAD and ACS patients [15].
Zeng et al.	2023	Prospective cohort	7662 ACS patients	January 2015 and May 2019	>1 year mortality	61.0 ± 10.6 years	3179 patients with DM	Hyperglycemia was quantified by SHR. SHR was calculated using standard formula. Patients were further divided into three group based on SHR value, i.e., Group 1 (SHR ≤ 0.84), Group 2 (0.84 -1.10), and Group 3 (SHR>1.10) [16].	Group 3 of SHR was strongly associated with all‐cause mortality (HR 1.80, 95% CI= 1.29–2.51) in both diabetic and non-diabetics. Only the third SHR group (SHR >1.10) was associated with heightened risk of all‐cause mortality (HR 1.72, 95% CI 1.09–2.71) in patients with diabetes. Both the second (0.84 -1.10) and third group (>1.10) were associated with elevated risk of all‐cause mortality (G2 vs. G1: HR 1.63, 95% CI 1.01–2.62; G3 vs. G1: HR 1.66, 95% CI 1.02–2.73) in patients without diabetes [16].	Results suggested that high SHR was independently associated with long‐term poor prognosis in ACS patients with or without DM [16].
Zhang et al.	2024	Prospective cohort	3812 ACS patients with TVD	2720 patients at were recruited between 2004 and 2011 at Fuwai Hospital and those at 1092 patients were recruited at Dalian Medical University between 2013 and 2018.	>1 year mortality	61.9 ± 10.3 years	1949 patients with DM	The levels of blood glucose and HbA1con admission were measured. SHR was calculated as follows: admission blood glucose (mmol/L)/ {1.59 × HbA1c(%) − 2.59}. The study participants were stratified into the following five SHR groups in increments of 0.25: low SHR (SHR < 0.50, n = 113), median-to-low SHR (SHR 0.50 to < 0.75, n = 1154), median SHR (0.75 to < 1.00, n = 1730), median-to-high SHR (1.00 to < 1.25, n = 519), and high SHR (SHR ≥ 1.25, n = 285) [17].	Patients with high SHR had an increased risk of cardiovascular mortality in comparison with the median SHR as shown by multivariable cox regression analysis, after adjustment for confounding (patients with high SHR vs. median SHR: unadjusted HR 2.049, 95% CI 1.325–3.168, P = 0.001; adjusted HR 1.809, 95% CI 1.160–2.822, P = 0.009) [17].	High SHR is associated with the long-term risk of worse cardiovascular outcome in TVD patients with ACS [17].
Zhao et al.	2017	Cross-sectional	10538 AMI patients	NA	NA	67 years	2280 had DM history	Admission blood glucose level was measured on day 0 or day1. Four group were formed based on admission glucose level, i.e., hypoglycemia (admission glucose <3.9 mmol/L), euglycemia (3.9–7.7 mmol/L), moderate hyperglycemia (7.8–11.0 mmol/L), and severe hyperglycemia (≥11.1 mmol/L), according to the diabetes mellitus (DM) guidelines [18].	Among patients who were diabetic, hypoglycemia (OR = 3.02, 95% CI [1.20–7.63]), moderate hyperglycemia (OR 1.75, 95% CI[1.04–2.92]), and severe hyperglycemia (OR 2.97, 95% CI {1.87–4.71}) were associated with increased risk for mortality, but among non-diabetic patients, only moderate hyperglycemia (OR 2.34, 95% CI {1.93–2.84}) and severe hyperglycemia (OR 3.92, 95% CI {3.04–5.04}) were associated with increased mortality risk [18].	Higher admission blood glucose predicted worse outcome in both diabetic and non-diabetics AMI patients [18].

Participant Characteristics

The total number of patients included in the study was 275061 while 62980 were the total participants of observational studies. Four studies were conducted specifically in STEMI patients, nine studies in acute myocardial infarction (AMI) patients, one study specifically in NSTE-ACS patients, three studies in ACS patients and one study in coronary artery disease (CAD) patients which included ACS patients as well. Among observational studies 17179 patients had diabetes mellitus (DM). Three studies were conducted in non-DM patients, while one study included 2389 pre-DM patients and one study divided DM into DM1 and DM2. Among review studies Alkatiri et al. had 1/3rd cases as DM while Cheng et al. included 24 studies containing DM cases [1,3]. Majority of cases in our studies underwent percutaneous coronary intervention (PCI).

Outcome

The main aim of this study was to review the association between acute hyperglycemia with short- and long-term mortality in patients with ACS. The expected outcome was an increase in short- and long-term mortality with increase in blood glucose level. Short-term mortality included in-hospital mortality and ≤ 30 days mortality and long-term mortality include >30 days to ≤1 year and >1 year mortality.

Quality Assessment

We used Newcastle-Ottawa scale for quality assessment of 18 observational stuides and Assessing the Methodological Quality of Systematic Reviews (AMSTAR)-2 scale for four systematic review and meta-analysis. Two cohort studies and two systematic reviews and mataanalysis were excluded due to higher risk of bias. The quality assesment tables for the included 16 observational and two systematic review and meta-analyses are presented below in Tables 4-6.

**Table 4:** Newcastle-Ottawa scale for cohort studies. Chen et al. [2], Ding et al. [4], Gencer et al. [5], Kumar et al. [6], Liu et al. [7], Paolisso et al. [8], Schmitz et al. [9], Shahid et al. [10], Thoegersen et al. [11], Upur et al. [12], Wei et al. [13], Xie et al. [14], Xu et al. [15], Zeng et al. [16], and Zhang et al. [17].

Questions	Chen et al.	Ding et al.	Gencer et al.	Kumar et al.	Liu et al.	Paolisso et al.	Schmitz et al.	Shahid et al.	Thoegersen et al.	Upur et al.	Wei et al.	Xie et al.	Xu et al.	Zeng et al.	Zhang et al.
1. Was the exposure and outcome of interest clearly explained?	Yes	Yes	Yes	Yes	Yes	Yes	Yes	Yes	Yes	Yes	Yes	Yes	Yes	Yes	Yes
2. Did they mention exposed people clearly in the local area?	Yes	Yes	Yes	Yes	Yes	Yes	Yes	Yes	Yes	Yes	Yes	Yes	Yes	Yes	Yes
3. Did they mention non-exposed people clearly?	No	No	No	Yes	No	Yes	No	No	No	Yes	No	No	Yes	No	Yes
4. Demonstration that outcome of interest was not present at start of study?	Yes	Yes	Yes	Yes	Yes	Yes	Yes	Yes	Yes	Yes	Yes	Yes	Yes	Yes	Yes
5. Were the people similar (other than exposure)?	Yes	No	Yes	Yes	Yes	Yes	Yes	Yes	Yes	Yes	Yes	Yes	Yes	Yes	Yes
6. Were exposure and outcome measured in the same way in both groups?	Yes	Yes	Yes	Yes	Yes	Yes	Yes	Yes	Yes	Yes	Yes	Yes	Yes	Yes	Yes
7. Was follow-up long enough and sufficient enough for outcomes to occur?	Yes	Yes	Yes	Yes	Yes	Yes	Yes	Yes	Yes	Yes	Yes	Yes	Yes	Yes	Yes
8. Was the study published in an indexed journal?	Yes	Yes	Yes	Yes	Yes	Yes	Yes	Yes	Yes	Yes	Yes	Yes	Yes	Yes	Yes
Total (out of 8)	7	6	7	8	7	8	7	7	7	8	7	7	8	7	8

**Table 5:** Newcastle-Ottawa scale for cross-sectional study. Zhao et al. [18]

Questions	Zhao et al.
1. Were the sample representative of the cases?	Yes (1)
2. Sample size >400?	Yes (1)
3. Response rate > 95%?	Yes (1)
4. Validated screening/surveillance tool?	Yes (2)
5. The potential confounders were investigated by sub-group analysis or multivariable analysis.	Yes (1)
6. Assessment of the outcome: a) Independent blind assessment, 2 scores; b) Record linkage, 2 scores; c) Self report, 1 score; d) No description, 0 score.	Self report (1)
7. The statistical test used to analyze the data is clearly described and appropriate. 1 score.	Yes (1)
Total (out of 9)	8

**Table 6:** AMSTAR-2 scale for systematic reviews and meta-analyses. AMSTAR-2 scale: Assessing the Methodological Quality of Systematic Reviews-2 scale, RoB: risk of bias, PICO: participants, interventions, comparisons and outcomes. Alkatiri et al. [1], Cheng et al. [3].

Questions	Alkatiri et al.	Cheng et al.
1. Did the research questions and inclusion criteria for the review include the components of PICO?	Yes	Yes
2. Did the report of the review contain an explicit statement that the review methods were established prior to the conduct of the review and did the report justify any significant deviations from the protocol?	Yes	No
3. Did the review authors explain their selection of the study designs for inclusion in the review?	Yes	Yes
4. Did the review authors use a comprehensive literature search strategy?	Yes	Yes
5. Did the review authors perform study selection in duplicate?	Yes	Yes
6. Did the review authors perform data extraction in duplicate?	No	No
7. Did the review authors provide a list of excluded studies and justify the exclusions?	Yes	Yes
8. Did the review authors describe the included studies in adequate detail?	Yes	Yes
9. Did the review authors use a satisfactory technique for assessing the RoB in individual studies that were included in the review?	Yes	Yes
10. Did the review authors report on the sources of funding for the studies included in the review?	Yes	Yes
11. If meta-analysis was performed did the review authors use appropriate methods for statistical combination of results?	Yes	Yes
12. If meta-analysis was performed, did the review authors assess the potential impact of RoB in individual studies on the results of the meta-analysis or other evidence synthesis?	Yes	Yes
13. Did the review authors account for RoB in individual studies when interpreting/ discussing the results of the review?	Yes	Yes
14. Did the review authors provide a satisfactory explanation for, and discussion of, any heterogeneity observed in the results of the review?	Yes	Yes
15. If they performed quantitative synthesis did the review authors carry out an adequate investigation of publication bias (small study bias) and discuss its likely impact on the results of the review?	Yes	Yes
16. Did the review authors report any potential sources of conflict of interest, including any funding they received for conducting the review?	No	No
Total	14	13

Acute hyperglycemia and mortality in ACS patients

We analyzed the different parameters used to measure acute hyperglycemia like SHR, ABG, and FPG and their raised level association with short- and long-term mortality in ACS patients.

Acute Hyperglycemia Defined by Stress Hyperglycemia Ratio

Seven studies used SHR as a parameter to quantify acute hyperglycemia. Four studies assessed association with short-term mortality (three in-hospital and one study <30 days). Studies like Chen et al. and Xu et al. for in-hospital mortality and studies like Schmitz et al. for < 30 days mortality confirmed statistically significant association of SHR with short-term mortality (p<0.05) [2,9,15]. Wei et al. found high SHR, ABG and FPG all to be associated with higher in-hospital mortality in STEMI patients [13]. Four studies studying the association of SHR with long-term mortality found the association to be statistically significant especially in non-diabetics (p<0.05).

Acute Hyperglycemia Defined by Admission Blood Glucose

Eight studies used ABG as a parameter to measure acute hyperglycemia. Eight studies studied the short-term mortality among which seven in-hospital and one < 30 days mortality. One study used more than year mortality as outcome. All seven studies studying in-hospital mortality found higher ABG to have statistically significant association with in-hospital mortality. Thoegersen et al. found increasing ABG to be associated with 30-day mortality in a dose-dependent manner [11]. Paolisso et al. found ABG to be associated with poor long-term outcome in both MIOCA (myocardial infarction and obstructive coronary arteries) and MINOCA (myocardial infarction with no obstructive coronary atherosclerosis) patients [8]. Alkatiri et al. found ABG and FPG to be associated with short-term mortality and glycosylated hemoglobin (HbA1c) to be associated with long-term mortality [1]. Cheng et al. also found significant association of ABG with short- and long-term mortality [3].

Acute Hyperglycemia Defined by Fasting Plasma Glucose

Fasting plasma glucose (FPG) was used by three studies as a parameter to measure acute hyperglycemia [5,12,13]. Upur et al. and Wei et al. studied in-hospital mortality and found FPG to have stastistically significant association with in-hospital mortality in ACS patients [12,13]. Gencer et al. found FPG >10 mmol/L to predict one year mortality in ACS patients [5].

Discussion

In this systematic review we reviewed altogether 21 articles from three databases (PubMed, Google Scholar, and Science Direct). Eight studies used ABG, seven used SHR, and three used FPG as a parameter for measuring acute hyperglycemia. Our main finding is that acute hyperglycemia quantified by SHR, ABG, or FPG are predictors of short-term and long-term mortality in ACS patients. Higher SHR has been shown to increase in about-hospital mortality in both diabetics and non-diabetics [15-17]. The proposed mechanism for acute hyperglycemia in ACS is (1) sympathetic activation and (2) hormonal influence. Sympathetic stimulation leads to release of norepinephrine leading to enhanced gluconeogenesis. Also glucagon is released due to action of norepinephrine and both lead to hyperglycemia [19-21]. Similarly, during periods of stress cortisol is released which also increases glucose level in blood. There may be pancreatic B cell dysfunction and tissue insulin resistance contributing to hyperglycemia [19,20]. Released epinephrine also mobilizes free fatty acids from adipose tissue which contribute to insulin resistance (IR) and decreases muscle glucose uptake [21].

Acute hyperglycemia has an added effect on prognosis of ACS and increases the worse effect of cellular damage caused by myocardial ischemia [20]. Although the precise mechanism on how hyperglycemia increases mortality in ACS has not been elucidated but there are certain changes that it brings like increase in free fatty acids increasing arrhythmia risk, increase in reactive oxygen species, decreased functioning of nitric oxide with consequent microvascular and endothelial dysfunction, increased infarct size, reduced collaterals, and vascular inflammation [20,21]. Also acute hyperglycemia increases the release of inflammatory and vasoconstrictive factors, increases QT interval,decreases ischemic preconditioning and increases no reflow [19]. Stress hyperglycemia in non-diabetic STEMI patients leads to increased incidence of cardiogenic shock, contrast induced nephropathy and mortality [21].

Association Between Acute Hyperglycemia and Short-Term All-Cause Mortality

Ten studies focused on in-hospital mortality and two studies focused on ≤30 days mortality. Chen et al. found SHR to be a significant predictor of short-term mortality especially in non-diabetics while Xu et al. found SHR to be associated with short-term mortality regardless of diabetic status [2,15]. Kumar et al. and Shahid et al. found that ABG as a single most significant predictor of poor in-hospital outcome in ST elevation-ACS regardless of diabetic status [6,10]. Ding et al., Paolisso et al. and Zhao et al. also found ABG as a significant predictor of in-hospital mortality in AMI patients regardless of diabetic status [4,8,18]. Liu et al. found incidence of death in NSTE-ACS patient without diabetes significantly increased with increased ABG [7]. Upur et al. found higher FPG at admission to be associated with increased mortality in AMI patients [12]. Wei et al. found ABG, SHR, FPG all to be associated with increased in-hospital mortality in patients with STEMI undergoing PCI [13]. Schmitz et al. found SHR and Thoegersen et al. found high admission blood glucose to be associated with increased short-term mortality in patients with ACS regardless of diabetic status [9,11]. Alkatiri et al. found ABG and FPG to be associated with short-term poor outcome in STEMI patients [1]. Cheng et al. found admission hyperglycemia to predict higher short-term worse outcome in AMI patients than long-term [3].

Association Between Acute Hyperglycemia and Long-Term Mortality

One study focused on one year mortality and six studies focused on more than one year mortality. Schmitz et al. and Zhang et al. found elevated SHR to be associated with higher long-term mortality; more so in diabetic than non-diabetic [9,17]. While Xie et al. and Zeng et al. found elevated SHR to be independently associated with long-term mortality irrespective of diabetic status [14,16]. Paolisso et al. found ABG to be an independent predictor of poor outcome in both MIOCA and MINOCA irrespective of DM status [8]. Upur et al. found FPG as an independent predictor of long-term mortality irrespective of diabetes [12]. Gencer et al. found FPG to be a strong predictor of one year mortality in non-diabetics with ACS [5].

Timely initiation of insulin and other glucose lowering therapy has been shown to decrease morbidity and mortality in ACS patients especially in non-diabetics [20]. What is suggested by most studies done till date is acute hyperglycemia in ACS patients is an independent predictor of morbidity and mortality regardless of diabetic status and that timely therapeutic interventions to control blood glucose level can reduce adverse impact of acute hyperglycemia [19-21]. Also the mechanism behind acute hyperglycemia leading to increased mortality in ACS patients is not precisely elucidated. This topic needs further research in area of cutoff value of acute hyperglycemia in diabetics and non-diabetics for starting therapeutic intervention. Our study may serve as backbone for further studies in future.

Limitations

We were able to review only 21 studies which is slightly less. Similarly we included only free full text articles and also included articles using SHR, ABG, and FPG only to quantify acute hyperglycemia. We conducted review of recently published articles only so that we could provide a up-to-date background on this topic.

## Conclusions

From our review, we came to a conclusion that acute hyperglycemia quantified by three important parameters, i.e., stress hyperglycemia ratio (SHR), admission blood glucose (ABG), and fasting plasma glucose (FPG) is a predictor of significant short- and long-term mortality in acute coronary syndrome patients. SHR predicted short-term mortality in both diabetics and non-diabetics while long-term mortality more in diabetics than in non-diabetics. ABG and FPG predicted short- and long-term mortality in both diabetics and non-diabetics. Certain things like the cutoff value for classifying acute hyperglycemia in both diabetics and non-diabetics still remain debatable and differ from study to study. So, further research needs to be done on that matter. We have systematically reviewed recent literatures that will definitely benefit learners and will provide a base for further research.
